# The Converging Lines of Medical and Surgical Treatment1President’s address, delivered at the meeting of the Central New York Medical Association, held at Buffalo, N. Y., June 2, 1891.

**Published:** 1891-08

**Authors:** Nathan Jacobson

**Affiliations:** Professor of Clinical Surgery and Laryngology, College of Medicine, Syracuse University, Syracuse, N. Y.


### THE CONVERGING LINES OF MEDICAL AND SURGICAL TREATMENT.[1]

By NATHAN JACOBSON, M. D.,
Professor of Clinical Surgery and Laryngology, College of Medicine, Syracuse University, Syracuse, N. Y.

The selection of a general subject as the text of an address implies the presentation of matters which have become trite by frequent repetition. It is not my purpose to laud the special work of the masters of our day, in medicine and surgery, nor to give expression to such optimistic views as did Boyer as long ago as 1814, when, impressed by the achievements of the British surgeons, he wrote: "Surgery seems to have attained the highest degree of perfection of which it is capable."

The first great achievement of the antiseptic era was the perfection of operative procedures and the elimination thereby, in the course of wound repair, of many incidental features which previously had been regarded as unavoidable. Emboldened by the security thus furnished, the surgeon next invaded new territory. New operations were undertaken, and the legitimate field of surgical practice was vastly extended. Cavities of the body, regarded as too sacred for invasion, were opened. Organs formerly considered essential to our economy were removed with impunity. During this antiseptic era, the study of the pathogenic organisms has received new impetus. When, in November, 1875, Robt. Koch brought his cultures of the anthrax bacilli to Breslau, and placed them before Cohn and Cohnheim, the latter, overwhelmed with the importance of his work, and the simplicity, yet exactness, of his methods, prophesied that Koch would yet astonish the world with future discoveries. The verification of the prophecy was not long delayed. With the cultivation of microbes upon solid media, with the isolation of the bacillus of tuberculosis and the specific micro-organisms of other diseases, surgical pathology assumed a new phase, and many of its chapters had to be rewritten. This better appreciation of certain processes has materially changed the relationship of surgeon and physician to each other.

There is, today, no question of greater importance than that which concerns the proper line of limitation of medical and surgical practice. There are diseases running but a brief course, yet so violent and serious as to destroy life within a few days, diseases

1. President's address, delivered at the meeting of the Central New York Medical Association, held at Buffalo, N. Y., June 2, 1891.

which the physician, by sad experience, has learned are entirely beyond the reach of medical aid, but which now, with a better conception of their pathology, are, if seen sufficiently early, often stayed by surgical procedures. There is another class, not of this fulminating type, but which pursues a chronic course, persistently eludes medicinal treatment, and which should be relegated to the surgeon's care. The invasion of the cranial cavity, in the pre-antiseptic era, was much dreaded because of the resulting inflammatory reaction which so often terminated fatally. While Lister was working in the wards of the Glasgow Royal Infirmary, establishing the principles of the proper treatment of wounds in other parts of the body, an earnest colleague, William Macewen, showed that cranial injuries, and, indeed, extensive cerebral wounds, kept aseptic, were quite as amenable to treatment. With the discovery by Broca that a particular area of the brain was the center of articular language, the misconception that the brain performed its duties as a whole, as do the kidneys or the liver, was made apparent. With the growing knowledge of cerebral localization, and the protection afforded by modern surgery, the brain no longer remains a *noli me tangere*. Extensive physiological study and surgical advancement have been required to establish the claim of the surgeon to treat many brain affections heretofore considered the exclusive property of the physician.

These cases demand the careful study of both physician and surgeon. The former may regard in a more hopeful light the outcome of a surgical procedure, which he may believe warranted, than the latter, who recognizes more thoroughly its real dangers. V. Bergman says that in the field of brain surgery, it has occurred to him more than once to be requested by colleagues of rare ability to perform operations which he has been obliged to decline. And so he published his recent work, not, as he says, to extend its domain, but rather to place upon it the necessary restrictions which his experience has taught him should be imposed. While Keen says that any lesion which produces intracranial pressure, which unrelieved would prove fatal, which can be localized and differentiated with reasonable certainty, granted it be accessible, should be removed, my experience has taught me that before deciding upon operation we must determine quite as certainly that the condition is within the reach of surgical relief. I am satisfied that the field of brain surgery demands the most judicious kind of conservatism. With the appreciation of the brilliant achievements on either side of the Atlantic, must be remembered some very disastrous failures, some of the saddest mistakes in diagnosis, and, consequently, treatment, which could possibly have been made. And so, while we recognize today that many clinical pictures have in the past been misinterpreted, and while we know that cerebral hemorrhage will not be controlled by medicinal agents, that pus will not be spontaneously evacuated from the cranial cavity, that the pressure symptoms occasioned by tumors will not cease while the growth remains, it is essential that the physician and surgeon should act in concert, and, having exhausted the influence of medication in suitable cases, resort to surgical aid only when satisfied that the condition is removable by, and only by, operation. As to the mutual relationship of physician and surgeon in that class of functional disturbances of the brain which endanger life, retard mental development, and destroy comfort and usefulness, I shall make no reference, as this subject has been entrusted to abler hands, from whom we will hear during this session.

In an article which appeared in the *Deutsche Medicinische Wochenschrift*, in January of the current year, Lembke makes the bold assertion that cases of exophthalmic goitre should be admitted to the surgical, and not to the medical, wards of a hospital. He was led to this conclusion, having operated two patients presenting the most serious symptoms possible of this affection, in both of whom favorable results followed extirpation of the goitre. This position is held by very few surgeons. Even Billroth objects to the removal of the entire gland, believing that it often occasions tetanus, and that it is difficult to avoid section of the recurrent nerve. Yet recent investigations show that some, if not all, symptoms are due to functional derangement of the thyroid. Indeed, in some cases the serious symptoms have disappeared, sometimes permanently and again temporarily, after minor operations upon the gland. When life is endangered by suffocation, when distressing symptoms exist which refuse to yield to medicinal treatment, there seems good reason to look for relief from operative measures. The treatment of acute pleuritic effusion is a subject over which there no longer can be any dispute. It has been my experience, I believe that I can say unexceptionally, to find prompt and permanent recoveries after aspiration. As soon as a serous effusion is diagnosticated, be it acute or chronic, it should be withdrawn. The very fact that most of these cases are of tubercular origin, it seems to me, emphasizes the necessity of this procedure.

The fever rapidly subsides, and the danger of subsequent pulmonary disease is greatly lessened. Purulent collections in the pleural cavity are now referred to the surgeon for treatment, but it is frequently a matter of great regret that their recognition comes so late. Great as has been the relief of empyema by simple incision, with or without subsequent drainage, or by resection of the ribs, as occasion demands, the surgical treatment of pulmonary diseases has not been so satisfactory. The greater dangers, the bolder procedures required, and above all the uncertain limitation of the diseases with the still more uncertain outcome of operative interference, has not led to the general adoption of pneumonotomy for draining pulmonary cavities, not to say anything of the more serious operation of pneumonectomy. The most favorable cases of pulmonary abscess for surgical treatment, Mr. Godlee thinks, are those due to pulmonary gangrene.

Before the surgeon can establish his claim to the treatment of these cases, better results must be obtained than appears in the table prepared by Bull, of Norway. In twenty-six cases of lung abscess collected by him and treated by incision and drainage, but four were cured and six improved, while of the others it is said that nine were relieved (these could not have been much improved, or they would have been classed with the other six) and seven were not benefited by operation.

The affections of no part of the body bring the physician and surgeon together more frequently than do those of the abdominal cavity. Some develop slowly, while others are of such an acute character that they threaten life from their very onset. T. Gaillard Thomas, in an able essay a few years ago, brought to the notice of the profession the fact that ascites many times appeared in women, as the result of pathological conditions, entirely removable by surgical procedures. Often it is a small growth mischievous by its compression, again a tubercular peritonitis.

Hepatic and biliary disturbances, when amenable to operative treatment, have yielded many times brilliant results. It does not seem to me that the dangers of obstruction by biliary concretions are sufficiently recognized by the general practitioner. Many times, and Harley says in the majority of cases terminating fatally, there has been neither jaundice nor paroxysmal pain. There is no doubt that each year many lives are sacrificed either because the impacted concretions remain unremoved, or ulcerating through the gall bladder or ducts produce fatal hemorrhage or peritonitis. The recommendation to knead the distended bladder and force its contents into the intestines, seems to me, may lead to serious trouble.

It is, however, the acute abdominal disturbances, obstructive, inflammatory and perforative, which require from their beginning the united attention of physician and surgeon. These troubles present themselves with such suddenness, and yet with symptoms which do obscure their real nature, that many times it may be questioned whether, when their diagnosis can be made with sufficient certainty, the favorable moment for operation has not passed. The technique of abdominal operations has been so thoroughly perfected that fear of entering this cavity has substantially disappeared. Not so much can be said for the differential diagnosis of acute intestinal obstruction. Yet, even here the past two years have witnessed a marked advance.

On the evening of April 21, 1889, I saw, with Drs. Heffron and Totman, of Syracuse, a babe five months old being nursed by its mother. On the morning of this date he was suddenly seized with severe colic, vomiting, and the rectal discharge of blood-stained mucus. Dr. Heffron discovered at his first visit a tumor in the right iliac region; no fever. Intussusception was diagnosticated. A large quantity of water was carried well up into the colon through a long rubber tube, and the tumor apparently disappeared, but the vomiting after each nursing and the bloody discharge from the rectum continued. The child cried incessantly. At our consultation in the evening the tumor was again found to be present and more elongated than before. The abdomen was very rigid. This time under chloroform the colon was distended with water, the child inverted, the intestines kneaded. The tumor again totally disappeared. Opiates were advised, and delay until morning seemed justified, but when the morrow came the condition was extremely grave. The pulse could no longer be counted; temperature, 103 degrees; the tumor was present and exhibited some evidences of crepitation about it. The vomiting had become stercoraceous; the bloody stools persisted, and the child passed rapidly away. The entire period of illness did not exceed thirty hours. At the autopsy the tumor was found to no longer occupy the ileocecal region, but to be located well over towards the splenic flexure of the colon. About twelve inches of large intestine was occupied by small gut, which had dragged up into the colon some of the omentum, as well as a little of the ascending colon. The adhesions between invaginat ed gut and its receptacle were so firm that no amount of irrigation with whatever degree of force could have secured the reduction. In fact, it was literally impossible to draw out the invaginated mass, and the specimen, as preserved, presents the reduction uneffected. General peritonitis was present. Cases of such acuteness, and at such an early period of life, certainly present but little to encourage an operation, yet without it death is inevitable. The medical journals are not barren of successful operations in really desperate cases. The importance of this subject is apparent, as Treves, in the preface of his work on Intestinal Obstruction, says that over two thousand individuals die annually in England from various forms of obstruction of the bowels exclusive of hernia.

Hatherly, in his presidential address before one of the branches of the British Medical Association in 1888, took the ground that peritonitis was not to be treated by section except it be evident that the disease originated in local mischief. As there is a growing conviction that with but very few exceptions general peritonitis is the outcome of a local disturbance, most cases of inflammation of this structure demand surgical aid.

The classic paper of Fitz, before the Association of American Physicians in 1886, presented, in a remarkably able and comprehensive manner, the pathology of inflammation of the vermiform appendix. The importance of his conclusions cannot be overestimated. It is evident that an appendix once inflamed is a very dangerous tenant to harbor in the body. At the session of the Freie Vereinigung der Chirurgen Berlins, held October 13, 1890, it was made evident that the German surgeons are much more conservative than are we. Israel, for example, restricts operative interference to but a limited class of cases. The claim was made that suppuration occurs infrequently, Gerhardt believing it to be absent in ninety-five per cent. of the cases. Much the larger number, it was stated, recover unoperated. Operation, we are told, exposes the patient to diffuse peritonitis, and, therefore, when no abscess can be felt, we are to await the appearance of grave symptoms, indicating a change for the worse, either a receding temperature, with increasing pallor and mild cyanosis, or a sudden rise of temperature with generalization of pain previously localized, increased vomiting, etc. The autopsies, which can usually be made after operations performed at this period, tell the sad tale that surgical aid has been too long delayed. Three times within the brief period of as many months I have been called to cases of perforative appendicitis complicated with general septic peritonitis, to find the patients entirely beyond operative relief. It requires no statistical tables, no figuring of percentages, to determine this question. I may have had an unfortunate experience, but it has been my ill-fortune to have never seen a recovery in this type of cases under the opium and rest treatment. When, on the other hand, McBurney presents a list of twenty-four operations done during the past two years for perforative appendicitis, with but a single death, the surgical treatment is most emphatically justified. The one point upon which all turns, is, of course, the early recognition of the trouble. Most of these cases die before the fifth day, which was the earliest period fixed by Willard Parker for operation. Now, we know that the third twelve hours of the sickness is the time to consider surgical treatment. The many able papers presented at the last meeting of our State Society upon this subject, lay down the principles for guidance both in diagnosis and treatment. One point, I believe, cannot be too emphatically stated, and that is the much greater importance of the local symptoms as against the general ones. We depend today too much upon the thermometer in gauging the seriousness of the disease. We are leaning, as it were, too heavily upon a fragile glass staff. Within ten days I had occasion to see during his last hours a young attorney of bright prospects, who throughout his illness of five days had an unvarying temperature of ninety-nine degrees, and yet the autopsy showed, besides the perforation due to a fecal concretion, a most severe septic peritonitis. The pulse is the better indicator of the seriousness of the condition. If we wait until it goes to and above 150, becomes feeble, compressible and flickering, we had better allow our patient to pass away undisturbed by surgical interference.

Successful as has been the early treatment of perforative peritonitis due to typhlitis, the operative treatment of intestinal perforations in typhoid fever has been most unfortunate. I have gone over with considerable care the recent literature of this subject which begins with the case of Luecke, operated in October, 1885, and have collected the records of some eight cases, but have yet to find the report of a single successful operation. The reasons for this sad result are self-evident.

Although I have occupied more of your valuable time than was my intention, I desire to consider another class of cases, usually unrecognized, and to which the surgeon in the light of recent experiences may lay some claim. I refer to primary tuberculosis of the kidney. Primary tuberculosis of the kidney is generally held to be a very rare affection. I believe it to be of much more frequent occurrence than is ordinarily supposed. Morris speaks of two varieties. In the disseminated form, miliary nodules are scattered through the kidney. This, he says, is usually part of a general malady. The second is a strumous pyelitis. Kuester improved upon the description of the later form by calling it caseous, and described the condition encountered as a yellow, cheesy infiltration in the calices and pelvis of the kidney. If this substance be scratched away the membrane is found to be ulcerated, and in the ulcer numerous gray granules are scattered.

To illustrate the clinical history, as well as the pathology of the disease, permit me to report a case :

F. B., *æt.* 25, married, grocery clerk, father died *æt.* 45, of consumption, otherwise family history clear ; referred to me by Dr. J. H. Coe, of Syracuse; admitted to St. Joseph's Hospital, February 1, 1891; never had had any venereal disease. Enjoyed good health until one year prior to admission into hospital. At this time urination became painful and frequent. The right lumbar region was the seat of much pain. These symptoms steadily increased in severity. He became exhausted after very slight exertion. Pain extended down to the right testicle ; at first paroxysmal, it soon became constant ; sometimes it would extend down the right leg. Beginning with November, 1890, each urination was attended with pain through the penis and at the glans. He reported that on December 1, 1890, he passed a stone one half the size of a pea. This was followed by two more, at intervals, of a week. The last was attended with quite a little hemorrhage. No physician ever saw the so-called calculi, and in the light of what is now known of the case, I think it probable that they were cheesy masses. From this date until his admission into the hospital, his suffering was severe and constant. The desire to urinate obliged him to expel the bladder contents every fifteen minutes, day and night. Some days he suffered intensely. There never was any pain in the left lumbar region. nor along the left ureter. No concretions were discharged while under my care. The urine was frequently analyzed, and it was found usually to be alkaline, with sp. gr. varying from 1008 to 1015, always albuminous, no casts, and though frequently sought for, no bacilli of tuberculosis were found, except in the urine discharged the day before operation. The urine persistently presented a stringy sediment upon standing, while the supernatant fluid remained clear. The morning temperature was, as a rule, normal. Each evening it rose to or above 100 degrees F. He was pale, lost some in flesh, and was growing constantly weaker. He had no cough nor other symptoms suggesting disease of the respiratory organs. Manipulation of the right lumbar region would always greatly aggravate his suffering, that of the left produced no disturbance. Physical examination: The urethra was clear and patent. The right lobe of the prostate was smaller than the left; both very firm. The sound revealed nothing abnormal in the bladder. Under ether, careful search for a tumor in the lumbar region failed to reveal any abnormal growth. The kidney could not be made out by palpation. February 20th, with Dr. H. L. Elsner, of Syracuse, the bladder was examined with the Leiter cystoscope. A slightly darkened area at the opening of the right ureter was the only abnormality apparent. The patient grew steadily worse. His suffering was so severe that he insisted that death would be preferable to his present condition, and implored me to relieve him by operation, if possible. I decided to do nephrectomy. The lumbar operation was performed March 23, 1891, with the assistance of the staff of St. Joseph's Hospital. When the kidney came into sight, its surface was found to be studded with small hard tubercular masses. The removal of the organ was quite difficult, as it was extremely long, reached well under the ribs, and tapered above to a point. Moreover, it was very friable, and was torn in the effort to remove it. Its capsule was also stripped off in its removal. The cavity was packed with iodoform gauze. Reaction was quite sharp; cystitis developed. The urine at first reduced greatly in quantity, reached the normal amount about the third day, but became very ammoniacal. By this time the general condition had decidedly improved, and the cystitis was really the only condition requiring much attention.

I will not delay you with a history of the case since the operation, more than to say that for the first month he steadily improved and was able at this time to be dressed and sit up during a part of each day. Since then his condition, however, has not been satisfactory. He does not gain strength nor weight, although his appetite is excellent. He still has an evening rise of temperature. He is not obliged to urinate oftener than once in two or three hours, and suffers no pain whatsoever. The urine examined on May 30th was found to be light yellow in color, some viscid sediment upon standing; sp. gr. 1012 alkaline, a small amount of albumin and a few bacilli present. I present to you the kidney for inspection. It is $5\frac{1}{8}$ inches in length, $2\frac{1}{2}$ inches in width, and $1\frac{1}{6}$ inches in thickness. Its surface is studded with millet-seed tubercles, especially marked at the upper pole. The cut surface is pale, the cortex swollen and granular. The calices contain gray ulcerations covered with cheesy pus. The ulceration is very marked just below the outlet of the pelvis.

Dr. Sears, pathologist to the hospital, made the microscopical examination. He reports that the cheesy masses contain great numbers of bacilli of tuberculosis. Thin sections of the kidney were found to contain a few bacilli, most numerous near the seat of ulceration, but extending up the straight collecting tubules. In other sections, the epithelium of the convoluted tubules was seen to be swollen, granular, in many places detached. The tubules situated in the pyramids of Ferrein showed less destruction of epithelium. The Malpighian tufts nearly filled the capsules of Bowman.

This case brings up the question of diagnosis and treatment of primary renal tuberculosis. The specimen presented, it seems to me, strongly suggests that the so-called two forms of tubercular disease of the kidney are in reality but different stages of one, or at least may coëxist. When the pelvis of the kidney is the seat of tubercular degeneration, and cheesy particles are carried off with the urine, the diagnosis upon microscopical examination should be much more readily made than when the tubercular deposit is in the cortical substance. The chemical examination of the urine will reveal a low specific gravity, acid or alkaline reaction and the presence of a small amount of albumin. If the microscopic examination rests with a search for casts and urinary deposits, evidently the true nature of the disease will remain undiscovered. Rovsing, of Copenhagen, in a recent work upon bladder inflammations, takes the advanced ground that no urinary analysis is complete without its bacteriological examination. The bacilli in urine can be discovered by the Ehrlich method. They are found to be very slender and somewhat elongated. They cannot be discovered in ammoniacal urine, as the bacilli then fail to take the stain. The character of the urine must be corrected not by the addition of chemicals to it after its discharge, but by the administration of suitable drugs to the patient. Sometimes it is wise to pursue the method of v. Bergman, who advises that the bladder be first irrigated, and, when cleansed, the kidney is to be kneaded and its contents forced into the bladder. The catheter is now again introduced and the discharge thus gained carefully studied.

The suggestion appeared a few weeks ago in the *Centralblatt für Chirurgie*, (Emil de Vos. No. 18, 1891,) that pure egg albumen be added to four times its weight of distilled water. A precipitate settles to the bottom of the tube and 10 c. c. of suspected urine is added to the supernatant fluid, thoroughly shaken and heated in a water bath at 150–160 degrees F. to its coagulation. If the urine contains much albumen, it requires simple boiling. Allowing it to stand, the bacilli are more readily found in the sediment precipitated.

But however examined, the finding of the tubercular bacilli in the urine is pathognomonic. Very often the bacilli are not discovered, and then our diagnosis cannot be as positively made. Palpation, in our case and in many others, by whatever method practised, is often disappointing. Where the healthy kidney has, because of its increased labor, become hypertrophied, in at least one case, a mistake occurred in determining which was the diseased organ. When the symptoms suggest disease of a kidney and the diagnosis lies between tuberculosis, stone and beginning tumor, abdominal section has been advised and undertaken by Tait, Thornton and others, for the purpose of determining the condition of both kidneys, and of performing nephrectomy at the same time if it is deemed advisable. The cystoscope will, no doubt, prove of great service in deciding as to the absence of tubercular cystitis, the limitation of disease to a single kidney and to which one, by observing the character of the urine spurting from each ureter, and also that very important question as to whether there is a second kidney, that the removal of the only existing organ may be prevented. The temperature should be closely studied ; daily exacerbation in a doubtful case is very suggestive. When primary renal tuberculosis has been diagnosticated, and the other organs have been found to be free from disease, when it is rapidly progressive and the patient's life is made unendurable by his suffering, with no outlook for any improvement, the question of operative interference must be considered.

Surgical literature is not rich in nephrectomies done for this affection, and the question as to its advisability is still an open one. If done at all, it should be performed early, and just here comes in the difficulty of its early recognition. Kuester diagnosticated the disease in one patient where it had existed but eight weeks. The danger of dying later of tuberculosis of some other organ, the possibility, or rather probability, that the other kidney is diseased, render the operation anything but hopeful, and yet Martin reported a case in which the patient was alive and well eight years after operation, and successful cases were reported by Madelung, Koenig, v. Bergman, Israel, Miculicz and others, at the German Surgical Congress of 1890. But I am occupying too much time. Many valuable contributions are awaiting a hearing. It is evident, however, that the relations of physician and surgeon have decidedly changed during the last decade. There exist in almost every region of the body affections which demand the careful, earnest study of both, and only by this coöperation can we reach a conclusion as to the proper limits of medical and surgical treatment. No doubt, many surgical procedures now undertaken will be found involving too great danger with too little promise of benefit, and will, therefore, be abandoned, while other affections even now considered hopeless will be relieved by surgical measures. We are passing through a most critical period, and the good judgment of the profession will be greatly taxed in carefully analyzing the results of medical and surgical treatment, and in selecting that which is for the best.

